# Carbon-Based Quantum Dots for Supercapacitors: Recent Advances and Future Challenges

**DOI:** 10.3390/nano11010091

**Published:** 2021-01-03

**Authors:** Fitri Aulia Permatasari, Muhammad Alief Irham, Satria Zulkarnaen Bisri, Ferry Iskandar

**Affiliations:** 1 Department of Physics, Faculty of Mathematics and Natural Sciences, Institut Teknologi Bandung, Jalan Ganesha 10, Bandung 40132, Indonesia; fauliap@students.itb.ac.id (F.A.P.); aliefirham@student.itb.ac.id (M.A.I.); 2 RIKEN Center of Emergent Matter Science, 2-1 Hirosawa, Wako, Saitama 351-0198, Japan; 3 Research Center for Nanosciences and Nanotechnology, Institut Teknologi Bandung, Jalan Ganesha 10, Bandung 40132, Indonesia

**Keywords:** carbon, quantum dots, quantum capacitance, supercapacitor

## Abstract

Carbon-based Quantum dots (C-QDs) are carbon-based materials that experience the quantum confinement effect, which results in superior optoelectronic properties. In recent years, C-QDs have attracted attention significantly and have shown great application potential as a high-performance supercapacitor device. C-QDs (either as a bare electrode or composite) give a new way to boost supercapacitor performances in higher specific capacitance, high energy density, and good durability. This review comprehensively summarizes the up-to-date progress in C-QD applications either in a bare condition or as a composite with other materials for supercapacitors. The current state of the three distinct C-QD families used for supercapacitors including carbon quantum dots, carbon dots, and graphene quantum dots is highlighted. Two main properties of C-QDs (structural and electrical properties) are presented and analyzed, with a focus on the contribution to supercapacitor performances. Finally, we discuss and outline the remaining major challenges and future perspectives for this growing field with the hope of stimulating further research progress.

## 1. Introduction

**Figure 1:**
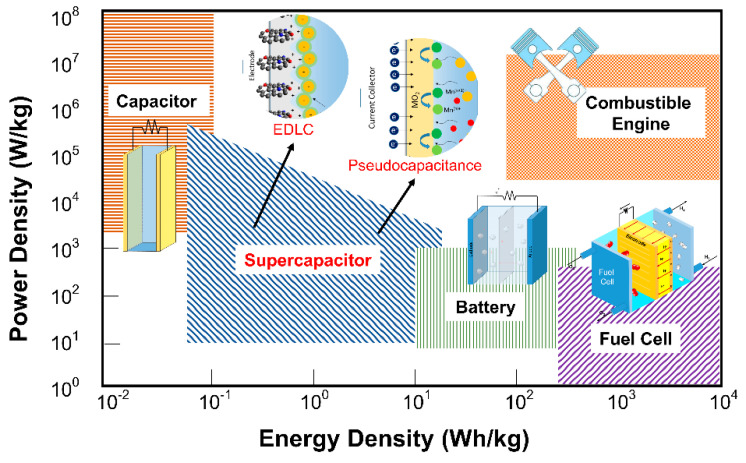
Ragone plot for several types of electrochemical energy storage devices: adapted from Reference [5]. Copyright American Chemical Society, 2004.

Fossil fuel shortages and environmental concerns regarding its uses for energy generation are among the most severe challenges in achieving sustainable development. Therefore, pursuits for alternative energy sources and energy storage devices are essential. Several critical parameters need to be achieved by those alternative energy storage devices, including high energy density, high power density, long lifecycle, environmental safety, and low cost [1,2,3]. To meet these critical parameters, the development of electrochemical-based energy storage devices (i.e., batteries and supercapacitors) still possesses fundamental research challenges, even though the earliest electrochemical supercapacitor patent was filed in 1957 by General Electric [4]. The Ragone plot (Figure 1), utilized as a figure-of-merit of energy storage devices, shows that the supercapacitor performance lies between those of conventional capacitors and batteries. A Supercapacitor (SC) provides a higher energy density and higher power density than batteries (>10 kW kg^−1^) and longer lifecycles (>10^5^ cycles) than a traditional capacitor [5]. Therefore, supercapacitors hold great prospect as ubiquitous energy storage devices for future electronic systems which requires high power and energy density yet durability and fast charge/discharge (in seconds).

Hybrid electric vehicles (EVs), memory backup systems, and portable electronic devices are demanding electronic systems [6,7,8]. In those systems, SCs can be integrated with primary high-energy batteries or fuel cells to provide temporary energy storage devices with a high power capability. Also, SCs could overcome the battery problem in high cycle rate operations. Nowadays, batteries are the primary energy storage for EVs. Nevertheless, in high cycle rate operations, batteries significantly heat up, which raises many safety concerns. On the other hand, high cycle rate operations also significantly decrease the capacity of batteries. These situations are in contrast with SCs that usually have significantly better cycling performance and durability for high rates of operations. Although it has many advantages and a larger power density, an SC still has a lower energy density than a battery. It is the real major challenge for the development and applications of SCs. We should overcome this problem by employing composite, doping, or other surface enhancements into materials.

In general, an SC comprises two electrodes that are immersed in an electrolyte. It may be electrically isolated by a separator, which can also play an essential role in SC performance. Based on the storage mechanism, SCs are classified into three types, i.e., the electric-double-layer capacitors (EDLCs), pseudocapacitors, and hybrid supercapacitors [9,10].

EDLC is the conventional supercapacitor in which capacitance arises solely from electrostatic charge accumulation between ions at the electrode/electrolyte interface [11]. This mechanism allows infinite time charge/discharge and is thus stable in the viewpoint of lifecycle stability. However, EDLCs suffer lower energy density than pseudocapacitors owing to limited specific surface area and the compatible electrode/electrolyte [12,13]. The most common electrode materials used in EDLC are porous carbon and its derivatives with large specific surface areas [14]. On the other hand, a pseudocapacitor relies on the reversible faradaic redox originating from the electroactive phases at the electrode/electrolyte interface. Even though it possesses high specific capacitance, a pseudocapacitor suffers challenging cycling stability because of its reversible faradaic redox process in the potential window [8]. Metal oxides, notably transition metal oxides, and conductive polymers are among the most common materials used in pseudocapacitors [15,16]. Another type is the combination of both the EDLC electrode and pseudocapacitive electrode in a single device with an intermediate SC performance. This device is a so-called hybrid supercapacitor. Based on the abovementioned energy storage mechanisms, numerous approaches have been extensively investigated to explore many different materials suitable for SC elements. Those approaches include nanostructuring and functionalizing the known electrode materials, designing materials by considering the energetic mismatch of electrolytes/electrodes, and exploring newly emergent materials [17].

Carbon-based Quantum Dots (C-QDs) emerged as materials that gained enormous scientific interest because of their unique properties [18,19]. This class of materials was firstly reported by Sun et al. in 2006 as carbon dots, owing to its nanometer-size diameter [20]. The carbon dots that were prepared from carbon nanotubes exhibited bright and colorful photoluminescence. These intriguing optical properties emerge from the quantum confinement effect, which typically occur in C-QDs with a diameter of around 10 nm [21]. However, some reports claimed that the quantum confinement effect was observed from carbon-based QDs with a larger diameter but less than 20 nm [22]. It should be noted that the occurrence of quantum confinement in this system is related to the significant increase in surface-to-volume ratio by reducing the C-QD diameter. The complex formation and structure of carbon-based QDs, which still largely remain unclear, make explanations of these material properties constrained and still debatable.

To date, several excellent review articles on the application of carbon-based QDs in energy conversion and storage applications, including solar cells, battery, thermoelectric devices, and supercapacitor, are reported [23,24,25]. However, they focus on the synthesis and the recent progress of C-QDs in applications broadly. To best our knowledge, there is a distinct lack of reviews mainly focusing specifically on Carbon-based QDs in supercapacitor devices.

The outstanding properties of C-QDs, i.e., their tunable electrical and optical properties, high stability, and excellent biocompatibility, not only correspond to the quantum confinement occurrence but also are due to variations in their structure and surface passivation [26,27]. There are various methods of producing C-QDs with different unique properties that lead this class of materials to become prospective for many applications such as optical devices, photocatalysts, sensors, and biomedicine [21,27,28]. Among the application avenues where the use of C-QDs might be distinguishable is its potential for components in electric and energy storage devices such as batteries and SCs.

In this review, we provide comprehensive discussions regarding the progress and the prospect of C-QD supercapacitors. The first part of the review explains the basic principles of a supercapacitor, including the charge storing mechanism, and deliberations of the essential parameters that need to be satisfied for any material before application in SCs. Subsequently, the general classification of C-QDs, their properties, and their preparation are summarized systematically. Based on the structural properties, the terminology of C-QDs can be categorized into three different types, i.e., Carbon Dots (CDs), Carbon Quantum Dots (C-QDs), and Graphene Quantum Dots (GQDs). The electrical properties of C-QDs are elaborated thoroughly for use in SC applications. Then, we summarize the recent progress of C-QD supercapacitor developments, either using bare C-QDs or its derivative to construct excellent EDLC materials. We also sum up the use of C-QDs as performance-enhancing agents for pseudocapacitance materials like transition metal sulfide, transition metal oxide, and conductive polymer. Considering the excellent properties of C-QDs, we provide critical insight into the challenges and the future development and application of C-QDs in energy storage devices.

## 2. Fundamental of Supercapacitor

### 2.1. Electric Double Layer Capacitor

**Figure 2:**
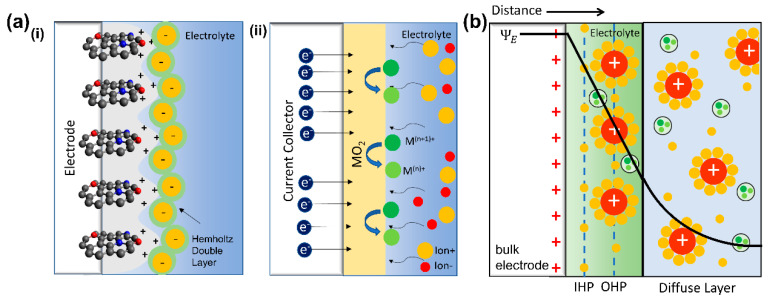
(**a**) Illustration of the Stern model for electrolyte ions on a charged electrode in (**i**) an electric-double-layer capacitor (EDLC) and (**ii**) a pseudo capacitance supercapacitor, as well as (**b**) a schematic model of ion distribution and mechanism.

Based on the electrical charge storage mechanism, supercapacitor devices are classified into three types: electric-double-layer capacitors (EDLCs), pseudocapacitors, and hybrid supercapacitors that combines both EDLCs and pseudocapacitors. Schematically, the mechanism behind each supercapacitor is shown in Figure 2a. EDLCs work via a phenomenon in which a charged object is placed inside a liquid. In the SC case, the liquid is an electrolyte, and the item is the electrode, which can also be a carbon-based material. The charges are stored electrostatically in the form of space charge accumulated at the electrolyte/electrode interface due to electric-double-layer formation. EDLCs can also be formed when a semiconductor is interfaced with a liquid electrolyte [29]. There are several models to simulate interface phenomena between charged electrodes and electrolytes. Helmholtz model is the simplest approximation to model charge distribution at a metal–electrolyte interface. The Helmholtz layer model is a well-known approximation and is the simplest model to explain charge distribution on the electrode/electrolyte interface [30]. This theory explains that the surface charge is neutralized by the opposite counterion situated at the surface. However, some phenomena are hardly explained with this theory alone since no rigid layers are formed on the surface, as described by this theory.

In 1913, Gouy-Chapman came up with a better model. This theoretical model assumes that the opposing counterions are not rigidly attached to the surface but tend to diffuse into the liquid phase. The thickness of the resulting double layer is affected by the kinetic energy of the counterions. This model is named the diffuse double layer model. Boltzmann distribution is used to model the counterion distribution near the charged electrode [31,32]. Furthermore, Stern proposed a better model by modification of the Gouy-Chapman diffuse double layer model using finite ion sizes. In this modified diffuse double layer model, the first ion layer is not precisely at the surface, but it has a finite distance from the surface. The surface may also adsorb some ions in the plane to form another layer, which is now commonly known as the Stern layer [33]. The ions are absorbed by the electrode and form layers which can be distinguished into Inner Helmholtz Planes (IHPs, for specifically adsorbed ions) and Outer Helmholtz Planes (OHPs, for nonspecifically adsorbed ions) (Figure 2b). Overall, the Stern model combined the concepts of the previous two models. This current model of the EDLC extends to many fields and provides better approximations to the experimental results [10,34,35].

To date, many theories and simulations have been developed to understand better and to estimate the capacitance values of the electric-double-layer capacitors (EDLCs). In general, EDLCs can be assumed as a parallel-plate capacitor so that its capacitance can be approximated using the following equation:

(1) $$C=\frac{\varepsilon_{0}\varepsilon_{r}A}{d}$$

where *A* is the surface area of the electrode, and *d* defines the effective thickness of the electric double layer (the Debye length). However, electrodes based on nanomaterials (e.g., nanowires, nanoparticles, nanotubes, quantum dots, etc.) have a high volume-to-surface-area ratio and different shapes of surface area. Consequently, another formulation is needed to rationalize the surface area of shapes and the formed pores. For example, Wang et al. modeled a Stern layer inside spherical pores, and the capacitance of the EDLC can be approximated as

(2) $$C_{SSA,me}=\frac{\varepsilon_{0}\varepsilon_{r}}{d}\left(\frac{R_{\mathrm{outer}}+d}{R_{\mathrm{outer}}}\right)$$

in which *R*_outer_ and *d* define the average pore radius and the electric-double-layer thickness, respectively. Using the abovementioned theoretical approach, we can rationalize that EDLC can be improved to possess a significantly higher capacitance value by increasing the electrode and electrolyte interface’s specific surface area. Nevertheless, there is still an incomplete understanding of the real charge distribution on nanopore electrodes, although the available models explain how EDLCs store energy [36].

**Figure 3:**
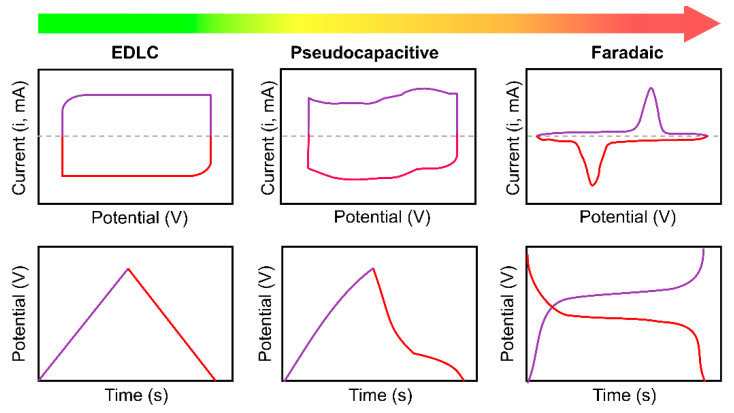
Schematic cyclic voltammograms and corresponding charge-discharge curves of electric-double-layer capacitor (EDLC), pseudocapacitor, and faradaic-type materials.

In an ideal EDLC, there are no faradaic and redox reactions on the electrode surface, which can be inferred from its cyclic voltammetry (CV) characterization, as shown in Figure 3 [37]. Without these electrochemical reactions, EDLCs have a more excellent lifecycle and are capable of a higher operation rate than pseudocapacitors. However, they have lower energy densities than pseudocapacitors. Electrochemical Impedance Spectroscopy (EIS) measurement is usually used to characterize SCs. An ideal EDLC shows a vertical curve representing a capacitor in the equivalent circuit [38,39].

In EDLC electrodes, a high specific surface area (SSA) is an essential parameter that determines the ion accessible area. To enhance the surface area of the EDLC electrode, increasing the size of the 3D building blocks and increasing the porosity of the materials are among the options [40,41]. However, if the porous size is less than the critical value (i.e., approximately 0.7 nm), most electrolytes would not be able to access the electrode surface [42]. Then, carbon-based materials like carbon nanofibers [43], activated carbon [44], and few-layer graphene [18] are other carbon allotropes promising for EDLC supercapacitor electrodes [44] owing to their large surface area. A significant capacity improvement could be achieved easily by controlling the interlayer interaction [45,46,47,48]. The control can be performed either by performing exfoliations (physical or chemical) to obtain thinner few-layer graphene (FLG) or by tuning the interlayer distance so that the ions can effectively intercalate [45,46]. For many other carbon-based and carbon-assisted materials that demonstrated preferable parameters for EDLC electrode applications, further studies are necessary. In particular, the other parameters, such as electrical conductivity and ionic intercalation capabilities, have not yet been thoroughly investigated to realize better performance. Enhancing the electrical conductivity in those materials might be achieved by optimizing elemental doping (nitrogen, sulfide, and phosphorus) into the materials in addition to the possibility to provide additional pseudocapacitance [47,48].

### 2.2. Pseudocapacitor

Pseudocapacitors store charges using redox or faradaic reactions that involve high-energy electrode materials, as shown in Figure 2a. The term pseudocapacitance arose due to this chemical reaction, which provides a fast and reversible redox reaction. Although the reaction is less similar to the reaction in battery materials, pseudocapacitance can be distinguished by the quick redox reaction on the surface or near-surfaces of electrodes [49]. The reaction mechanism of the pseudocapacitive system is illustrated in Figure 2b, which is predicted to fill the gap between EDLCs and batteries. A broader voltage window is allowed in pseudocapacitance since the surface redox mechanism is mainly controlled by its charge storage mechanism [50]. Combining pseudocapacitive materials, conductive materials, and the capacity from its EDLC performance is common in SCs [44,51].

Cyclic voltammetry (CV) is a standard method for determining the electrochemical properties in SC; therefore, it can distinguish the behaviors of different types of SCs. A typical CV measurement of pseudocapacitors (Figure 3) shows a broad redox peak that exhibits small peak-to-peak separation instead of a rectangular-like shape. The broad redox peak is proof of the reduction process that happens inside pseudocapacitive materials. At the same time, the rectangular-like CV characteristics are typically found in SCs with exclusively electrostatic processes, such as in EDLC [52,53].

Both EDLCs and pseudocapacitors face similar crucial challenges in increasing energy density for energy storage applications. In the capacitor, the energy stored inside is determined by capacitance and voltage, as $E=CV^{2}/2$. We can increase the stored energy density effectively either by improving the capacitance of the materials or by increasing the cell voltage [12]. Increasing the cell voltage of pseudocapacitive materials can be achieved by changing the utilized electrolyte. Since the performance of SCs would also depend on the utilized electrolytes, their selection and optimizations of the compatibility between electrodes and electrolytes are very crucial [54]. For instance, since an ionic liquid electrolyte has a wider electrochemical stable potential window (ESPW), the right composition with an electrode is proven to highly increase the energy density in a carbon-based electrode. By this outcome, it is possible in future development that an SC will overcome batteries’ energy densities (>100 Wh/kg) [55]. In electrolytes, ionic mobility and conductivity are essential parameters for suitable electrolytes in supercapacitors. Nevertheless, here, we do not review the electrolyte factors deeply.

The electrolyte and electrode are the two crucial parts that affect the SC directly. Conductive polymers, metal chalcogenides, and transition metal oxides are the most common materials used for pseudocapacitance SC electrodes. Their wide voltage window and high specific capacity values are supposed critical factors in high pseudocapacitance SC performances [56,57,58,59,60,61,62]. Various methods are used to synthesize SC electrode materials, such as sol-gel, electropolymerization/electrodeposition, in situ polymerization, vacuum filtration technique, chemical vapor deposition (CVD), co-precipitation, hydrothermal, and others. Since pseudocapacitive performance depends on the materials’ structures and properties, optimizing the parameters of various synthesis methods is vital [8,52,63,64].

Numerous reports of transition metal oxides and chalcogenides for pseudocapacitance SC electrodes have been published. However, they still suffer low conductivity or high internal resistance that are detrimental to the SCs’ high cycling rates capabilities. A composite of these pseudocapacitive materials with carbon-based materials is one of the best options to overcome these issues [65,66].

One of the examples is a composite of an established metal oxide or metal chalcogenide electrode materials with Carbon-based Quantum Dots (C-QDs). The as-prepared composites could exhibit enhanced electrical conductivity and other benefits for EDLCs or pseudo capacitance electrodes. Sahoo et al. combined NiS with carbon dots and demonstrated increased capacitance up to 880 F g^−1^ for the composite. The corresponding NiS was only limited below 770 F g^−1^ [67]. The use of carbon dots in the composite was found to increase the conductivity and to lower the electrolyte diffusion length during the charge–discharge process [67]. There are many more examples of composites combining C-QD and metal chalcogenides, which will be discussed in the latter part of this review.

Besides EDLC and pseudocapacitance, a new concept to increase capacitance in supercapacitor materials has emerged. Nanostructuring materials lead to modification of their electronic energy structure. Most notably, quantum confinement effects in 2-dimensional (2D) materials, 1-dimensional (1D) materials, and 0-dimensional (0D) materials give rise to the formation of abrupt changes in their electron density-of-states. In 1D materials, it is manifested by Van Hove singularity. On the other hand, quantum confinement in 0D semiconducting materials gives tunable energy bandgaps by particle size and form discrete quasi-atom-like energy levels. The ability to fill the van Hove singularity of 1D materials and the discrete energy level of 0D QD materials may lead to enormous capacitance. It is known as quantum capacitance [68,69,70]. This quantum capacitance is an essential factor that influences both the total capacitance and the EDLC performance [71].

## 3. Properties and Preparation of Carbon-Based Quantum Dots (C-QDs)

Carbon-based Quantum Dots (C-QDs) are one class of emerging nanomaterials that are mainly investigated for their photoluminescence properties. Surprisingly, C-QDs have also been reported remarkable electrochemical properties and could be applied for diverse energy storage applications. Thus, in this section, the structural and electrical properties of C-QD materials will be elaborated, especially the aspects attributed to energy storages device performances. Furthermore, the preparation of C-QDs directing the structural and electrical properties of C-QDs was also elaborated systematically.

### 3.1. Structural Properties

**Figure 4:**
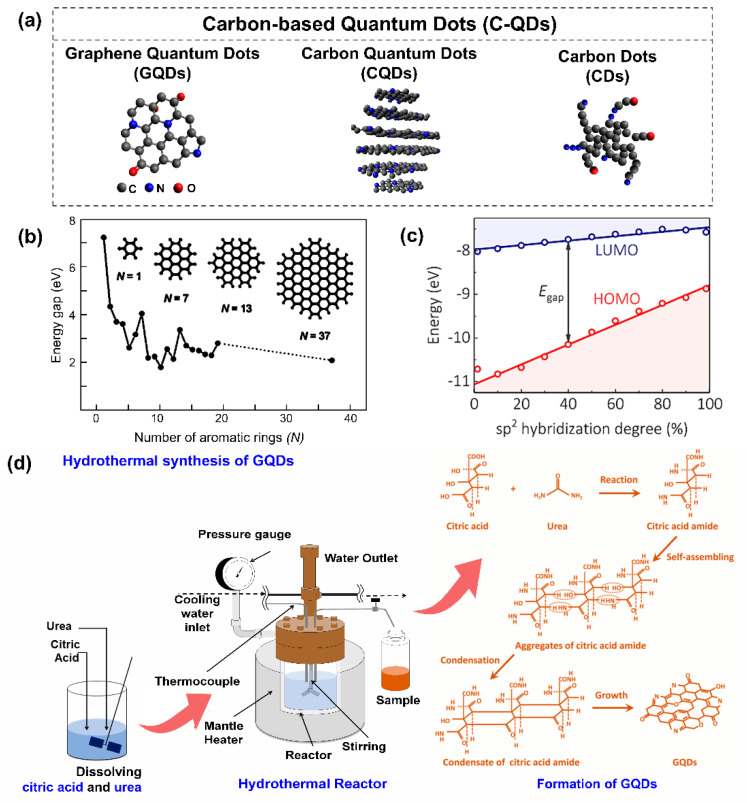
(**a**) Classification illustration for carbon-based quantum dots: adapted from Reference [72]. Copyright Royal Chemistry Society, 2016. (**b**) The energy gap of Graphene Quantum Dots (GQDs) as a function of the number of aromatic rings from a DFT calculation: reproduced from Reference [83]. Copyright John Wiley & Sons, 2010. (**c**) Highest occupied molecular orbital (HOMO)−lowest unoccupied molecular orbital (LUMO) energy gap of Carbon-based Quantum dots (C-QDs) as a function of the hybridization degree of the C−QDs’ domains: reproduced from Reference [76]. Copyright American Chemical Society, 2019. (**d**) The hydrothermal method for preparing the GQDs and the proposed mechanism of GQDs that were synthesized from citric acid and urea: reproduced from Reference [84]. Copyright Royal Chemistry Society, 2014.

Carbon-based QDs refer to a zero-dimensional fragment that was dominantly by carbon atoms [18]. Their size is on the scale of a few nanometers, and they exhibit some unique properties. In this review, the terminology C-QDs is used to describe all kinds of carbon-based materials that experience these size-dependent effects. These effects are either from the real quantum confinement effect or from the formation of electronic energy states due to emerging structures on the C-QD surface. Based on the complexity of their formation and structures, the terminology carbon-based QDs is categorized into three different terms, as proposed by Cayuela et al. (Figure 4a) [72]: carbon dots (CDs), carbon quantum dots (C-QDs), and graphene quantum dots (GQDs). These carbon-based QDs terms were classify based on the arrangement of carbon atoms, the crystalline structure, and dimensionality.

#### 3.1.1. Carbon Dots (CDs)

The term Carbon Dots (CDs) or Carbon Nanodots was proposed to identify the amorphous quasi-spherical nanodots that still lack quantum confinement [73]. Although CDs have a nanometer-size diameter in the range 1–20 nm, the quantum confinement phenomenon was not observed. Thus, the electronic bandgap of CDs did not strongly depend on its size. This term was also attributed to carbon-based polymer dots that exhibit typical photoluminescence properties in the range of nanometers [74]. CDs are mainly composed of an sp^3^ hybridization carbon core with a small domain of sp^2^ hybridization. In some cases, the X-ray diffractometer results of the CDs show the typical graphite peak centered at approximately 20° dominated by sp^3^ hybridization. Surprisingly, it was reported computationally that the hybridization degree of CDs plays a crucial role in bandgap engineering of CDs [75,76]. Thus, the optical and electronic properties of CDs rely on the surface functional groups that can easily be modified through a preparation method.

#### 3.1.2. Carbon Quantum Dots (C-QDs)

Carbon Quantum Dots (C-QDs) refer to spherical carbon nanosized with a crystalline structure and show the quantum confinement effect. C-QDs are a quasi-spherical particle with lateral and height size ranges of 1–20 nm [73]. The lattice constant of C-QDs lies between that of a graphene lattice and that of a graphite lattice [77,78,79]. However, the C-QDs have lower crystallinity than GQDs owing to the less crystalline sp^2^ carbon [80]. In most cases, C-QDs are strongly associated with surface passivation through functionalization or modification. The most common surface functionalization involves the oxygen-containing functional groups (e.g., carbonyl, carboxyl, and carboxyl) and nitrogen-containing functional groups (e.g., amine) [19,26,28]. Thus, both the quantum confinement effect and the surface functional groups contribute to C-QDs’ bandgap energy.

#### 3.1.3. Graphene Quantum Dots (GQDs)

Graphene Quantum Dots (GQDs) refer to 0D graphene sheets in a nanoscale dimension that exhibit strong quantum confinement [72]. Consequently, GQDs have good crystallinity with the lattice constant of graphene [18,81,82]. GQDs are dominated by the π-conjugate sp^2^ carbon structure, a fingerprint of polycyclic aromatic hydrocarbon molecules. Distinct from the pristine graphene, GQDs may consist of several layers of graphene sheets of 1–10 nm size. However, the mechanism of the edge site effect and the atomic doping (especially nitrogen) effect on GQDs have been considered similar to that of the graphene structure [81,83]. GQDs also could be enriched by the various surface functional groups as well as C-QDs.

Considering the SC’s electrode requirements, the edge site of the GQDs and the surface functional groups of all C-QD types enhance the SC’s performances. Specifically, the edge sites of GQDs could enhance the electric conductivity of GQDs owing to the doping effect of dangling bonds, the adsorption of ionic charges, and the formation of EDL capacitance [85,86,87]. Furthermore, these edge sites also enable co-synthesis of the C-QD and transition metals, which commonly uses an SC electrode to form robust metal-oxide-C (M-O-C) bonding. This bonding supports a higher electrical conductivity and a higher rate of the electrochemical process cycle [88]. The oxygen-containing surface functional groups –COOH, –COOR, and –OH induce water-soluble properties with excellent dispersibility [89,90,91]. These properties are beneficial in enhancing the wettability of the formed SC electrode, especially in water-based electrolytes [92]. Meanwhile, the nitrogen-containing surface functional groups on C-QDs, such as an amine-functional-group, were reported to improve SC’s pseudocapacitance performance [93]. Both edge sites and surface functional groups of the C-QDs are known to be easily modified during material synthesis, which will be discussed later.

### 3.2. Electronic Properties

The electronic transport properties of carbon-based QDs depend on the complicated interplays among representative electronic energy states of the carbon cores, the surface functional groups, the available heteroatom dopants, and the electronic couplings between neighboring nanoparticles [94,95]. Conventionally, efficient electron transfer is expected to originate from the arrangement of the high crystalline structure of carbon-based QDs that are free from any intrinsic defect. However, preparing C-QDs without any intrinsic defect is a tremendous challenge. Among the different types of carbon-based QDs, GQDs are the most promising for better electron transfer than CDs and C-QDs due to the higher degree of orders within their core. Furthermore, the well-defined and almost uniform edge sites of GQDs, formed by the size reduction of graphene, are beneficial for charge transfer between the carbon cores [96,97]. The edge states should assist in conduction due to the doping-like behavior. However, some of the functionalization of these GQD edge sites can be destructive. They may create electron trap sites, hindering electron transfer [87]. For example, oxygen functional groups are known to impede the electron transfer of GQDs due to disruption of the conductive sp^2^ carbon network. The structure of sp^2^ carbon increases accessible areas while improving conductivity. Zheng et al. reported that the hierarchical assembly of multi-diameter carbon nanotubes (CNT) was successfully synthesized as a supercapacitor. Moreover, the CNT provided a short ion diffusion path during migration in the charge/discharge process [98,99].

In C-QDs, the π-electron network that occurs from the sp^2^ hybridization may function as electron acceptors/donors, as a conducting medium for electron transport, or as bridges for electron transfer [73]. The use of C-QDs can be essential in preventing the decrease of electron conductivity after cycling due to cracking of the primary materials [100]. Kwon et al. reported that C-QDs have higher 2–4 electron mobilities times than its hole mobilities. Intrinsically, these properties might arise from a larger conduction bandwidth than the valence bandwidth. Those properties could be implemented for many optoelectronic devices and energy storage devices including supercapacitors. Moreover, a C-QD’s charge carrier mobility also depends on its ligand length, with longer ligand length resulting in a decrease in electron mobility exponentially [101].

Highest occupied molecular orbital (HOMO) and lowest unoccupied molecular orbital (LUMO) energies of GQDs are 5.3 eV and 3.8 eV below the vacuum [96]. In the structural design of GQDs, Eda et al. proved that the number of atomic rings inside GQDs determines the bandgap energies (Figure 4b) [83]. Otherwise, this bandgap energy can be tuned by several methods like adding dopants, changing the number of defects in the particles, functionalizing their surface, and engineering their size and shape. In C-QDs, the energy gap between π-states decreases as the number of aromatic rings increases [102,103]. This bandgap energy tuning capability provides pathways to utilize the quantum capacitance behavior of these nanoscale materials. Recently, a computational study of CD structures by the Rogach group reported that their bandgap energy depended on the hybridization degree from which the delocalized electron on CDs was attributed (Figure 4c) [76]. These delocalized electrons could have arisen from the large sp^2^ domains or the C–N configuration on the CD structure. Thus, the presence of a nitrogen atom on CDs could tailor both electronic properties and optical properties. It implies the potential uses of CDs, C-QDs, and GQDs for electronic device applications through bandgap engineering approaches.

### 3.3. Preparation of Carbon-Based Quantum Dots

Numerous reports on the preparation of C-QDs through various methods have been reported extensively. Generally, the preparation method of C-QDs is classified into two categories: “top-down” and “bottom-up” approaches. The top-down approach uses higher dimensional carbon-based materials, i.e., graphite, graphene oxide, and graphene, as starting materials to synthesize a nanoscale carbon structure via chemical or physical techniques. On the other hand, the bottom-up approach uses a smaller carbon-based molecule to generate the C-QD structure via chemical reactions.

#### 3.3.1. Top-Down Approach

The top-down approach includes the laser ablation, chemical oxidation, chemical exfoliation, arc-discharge, and ultrasonication methods [104,105,106]. In the laser ablation method, a nanosecond-pulsed laser with a specific wavelength is directed at a target. Dense plasma is obtained from the interaction between the laser and the target. The precursor solution and/or nitrogen, which serve as dopant atoms, induce a spontaneous plasma cooling process. The C-QDs are then produced through physical shearing of the carbon structure of the target and by incorporating the dopant atoms from the precursor solution. Nitrogen, oxygen, and sulfur doped on C-QDs were reported successfully prepared through this method [104,107,108]. The properties of the synthesized C-QDs strongly depend on the laser power, precursors, target type, and the concentration of the precursor solution. This method sufficiently produces C-QDs with high yield production and high purity. However, this method requires sophisticated equipment and a high-vacuum process, hindering upscaling of production for commercial needs.

Chemical-oxidation-induced synthesis is a successfully reported method that produces C-QDs with a controllable structure. Among all of the chemical oxidation agents, acidic-based oxidation agents have become the most commonly used due to their facile preparation [28]. As part of this method, a chemical exfoliation process occurs, exfoliating the stack carbon nanosheet into several carbon nanosheets through physical shearing. Then, the C-QDs are obtained from the reduction of these nanosheets. Although it produces well-controllable surface passivation of C-QDs, this oxidation-induced synthesis is time-consuming. A modified chemical or exfoliation process such as microwave-assisted reduction was then developed to accelerate the reduction process [109,110]. Electrochemical exfoliation involves both chemical oxidation and chemical exfoliation in the presence of the applied voltage. In brief, the anode of the carbon source is dipped in the electrolyte and is connected to the cathode. A voltage is then applied between the anode and cathode, which results in anodic oxidation of the electrolyte and anionic intercalation from the ionic liquid. The voltage allows the break-off of the carbon chemical bonding, resulting in an exfoliated carbon nanostructure. The most common carbon source is from large, graphitized carbon materials such as graphite, graphene, or graphene oxides [106]. This method is more environmentally friendly than the chemical and exfoliation processes due to the absence of potent acidic-based oxidation agents.

#### 3.3.2. Bottom-Up Approach

The bottom-up approach basically produces the C-QD structure from smaller carbon-based molecules through a chemical and physical reaction. This approach includes hydrothermal synthesis, microwave synthesis, and pyrolysis [84,111,112]. Among the many possible bottom-up approaches, the hydrothermal method has gained more scientific interest. This process involves water as a solvent or a dispersed medium for C-QD formations, which is beneficial for applications. Furthermore, this technique is preferable for large-scale production due to its ease of use, environmentally friendliness, and low cost compared to other preparation methods. Our group has developed various C-QDs from citric acid and urea as raw materials via a hydrothermal method since 2014 [81,84,111,113,114]. Based on a comprehensive analysis, the GQDs are formed by dimer or oligomer-sized aggregates of citric acid amide that condensate into nanostructure graphene-like sheets (Figure 4d) [84]. A further systematic study revealed that the carbon–nitrogen configuration on the GQD structure plays an essential role in controlling its optical properties. Thanks to the delocalized electron from pyridine-N and pyrrolic-N, the bandgap of the GQDs becomes narrow [81]. Reaction time, reaction temperature, hydrothermal heating rate, and raw material concentrations are key in controlling the formation kinetics of C-QDs [113]. Therefore, the optical properties of as-synthesized C-QDs could be tailored. It has been successfully applied as UV blocking layers [111] and in photothermal application [114].

In a microwave-assisted synthesis, a strong electromagnetic interaction between the precursor with the microwave irradiation leads to the formation of C-QDs. Several experimental parameters, i.e., microwave power, temperature, irradiation time, and solvent, are crucial in designing the desired C-QDs [24,25]. The different polarities of the utilized solvents determine the conjugation degree of sp^2^ carbons, which results in tailored energy gaps and structures [24]. Owing to abrupt heating due to microwave resonance, the as-synthesized C-QDs have broad size distributions and various surface functional groups. In the pyrolysis method, a carbonization process is the main component in forming C-QDs [112]. The starting material selection, reaction time, and temperature could be optimized to obtain the desired C-QDs. It was worth noting that all these bottom-up approaches require further purification steps to remove the by-product.

## 4. Recent Progress of Bare Carbon-Based Quantum Dots for Supercapacitors

**Figure 5:**
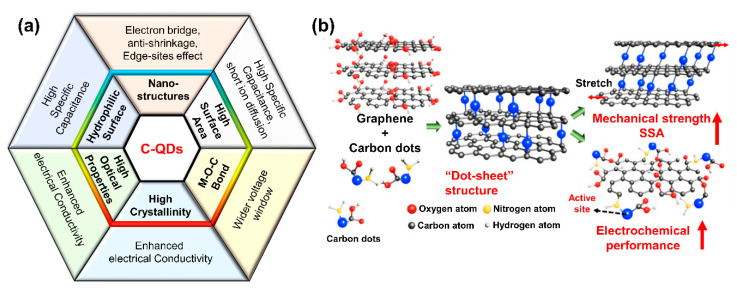
(**a**) A schematic showing the figure-of-merits of C-QDs composites for Supercapacitor (SC) applications that should be pursued and (**b**) a schematic illustration of the dot-sheet porous structure that consists of graphene and carbon dots: reproduced from Reference [115]. Copyright Royal Chemistry Society, 2018.

Owing to C-QDs’ superior properties, C-QDs can be applied in different types and elements of supercapacitors. Both electrode and electrolyte elements can utilize C-QDs as building blocks. This section summarizes the recent progress in the literature on the utilization of bare C-QDs as the electrode in supercapacitors. In general, C-QDs exhibit the capacitive behavior of an EDLC mechanism. However, recently, the use of C-QDs has also been reported to be beneficial for pseudocapacitors. Overall, we summarize the figure of merit of C-QDs in Figure 5a.

In bare C-QD SC electrodes, GQDs are more commonly used since they show good conductivity and edge-state-related properties that result in decent specific capacitance. On the other hand, the other types of C-QDs must be combined with high conductive materials to ensure high performance of the supercapacitor. Qing et al. introduced the combination of enriched carbon dots with graphene microfibers to produce high-performance supercapacitors [115]. The idea of combining CDs as nanofillers in graphene fiber is to enhance mechanical strength and to give a larger specific surface area (SSA). The specific capacitance demonstrated by CDs/graphene was as high as 67.37 μWh cm^−2^, three times larger than reduced graphene oxide (RGO) fiber. As shown in Figure 5b, the CDs formed a dot/sheet structure with the graphene to prevent any graphene restacking.

The CDs, which contain abundant functional groups, also contribute to the capacitance due to active electrochemical activity, which may lead to the existence of quantum capacitance [115,116]. In 2013, Wen-wen et al. reported the assembly of bare GQDs as an electrode of a micro-supercapacitor without additional electrode materials [117]. The GQDs used by these authors were synthesized using the solvothermal method with graphene oxide, prepared by the Hummer method, as a raw material. The as-synthesized GQDs had a spherical shape in the size range of 1.0−5.4 nm with a topographic height of 1.0−2.5 nm, indicating that most GQDs consist of 1−4 graphene layers. Through electrophoretic deposition, the GQDs were transformed into an electrode. The micro-supercapacitor was built by 32 in-plane interdigital Au microelectrodes that consisted of 16 positive and 16 negative microelectrodes. The microelectrode’s dimensions were 230 µm (width) × 10 mm (length) with the distance between microelectrodes at 200 µm. Surprisingly, the analysis results showed that the GQDs/GQD symmetric micro-supercapacitors had a good rate capability (up to 1000 V s^−1^), excellent power response with a small RC time constant (103.6 µs), high specific area (468.1 µF cm^−2^), and outstanding durability in 0.5M Na_2_SO_4_ aqueous solution. These excellent supercapacitance performances of GQD-based micro-supercapacitors were expected to strongly correspond to its superior properties of GQDs including the large specific surface area, the abundant active sites, and the accessible edges. These accessible edges are the hosts in the production of double layer capacitance on their surface [118].

**Figure 6:**
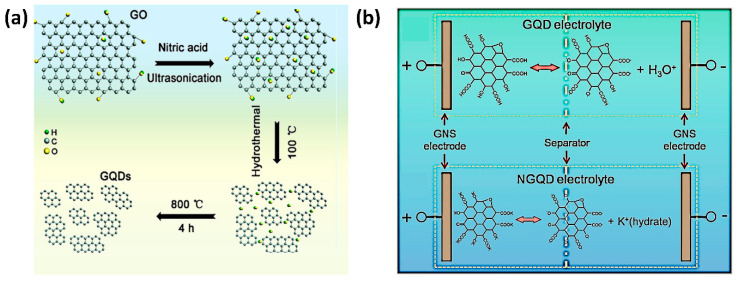
(**a**) Schematic of synthesis of GQDs with uniform size for an SC device: reproduced from Reference [119]. Copyright American Chemical Society, 2018. (**b**) Schematic Illustration of GQDs and an N-GQD solution as the electrolyte in EDLC supercapacitors: reproduced from Reference [120]. Copyright Springer Nature, 2016.

Due to their merit, GQDs are very attractive for constructing high-performance SCs with GQDs as its electrode [119]. Commonly, the GQDs, as in other C-QDs, were prepared by a hydrothermal or solvothermal method. In many cases, graphene oxide was chosen as the raw material, as illustrated in Figure 6a. Zhang et al. purified the GQDs by heat treatment to detach the oxygen-containing functional groups, which is known as hindering the electronic transport through the carbon core. The GQD electrode materials demonstrated an ideal electric double-layer supercapacitor with a specific capacitance value of 307.6 F g^−1^ at a scan rate of 5 mV s^−1^, a high energy density of 41.2 W h kg^−1^, and excellent capacitance retention after 5000 cycles. These excellent SC performances were attributed to the uniform nanoscale size (<5 nm) of GQDs that effectively restrain the restacking of graphene nanosheets and to the edge effect that improved the abundancy of the active site for ion diffusion [119].

Hypothetically, the GQDs could improve the conductivity of any electronic devices based on the superior properties of GQDs, including their high specific surface area, active edge site, and the C-QDs that preserve abundant free electrons in the system. However, this amine-enriched GQD electrode system still needs further investigation to clarify the electron transport mechanism [93].

Moreover, N-GQDs were also reported to demonstrate pseudocapacitive behavior [121]. The existence of pyrrolic-N/pyridine-N in N-GQDs is thought to be the origin of the capacitance increase that involves a reversible pseudocapacitive redox reaction and wider potential window [122]. The presence of sulfide, nitrogen, or oxygen as dopants in C-QD can result in the emergence of pseudocapacitive behavior. Ouyang et al. also reported that the trap states still exist even after doping the C-QD using sulfide [47].

Compared to other C-QDs, the use of GQDs in SC is widespread today. However, the use of GQDs for compositing is still primarily in its infancy, and its potential has not been fully utilized. Qing et al. reported the use of GQDs inside activated carbon to overcome the abovementioned issues. A similar phenomenon of the emergence of pseudocapacitive behavior from GQDs was also observed in GQDs embedded in activated carbon [123]. A performance comparison of the C-QD SC electrode is summarized in Table 1.

Su Zhang et al. revealed the potential application of GQD solutions as an electrolyte solution in the solid-state supercapacitor [120]. As shown in the Figure 6b, the GQDs were prepared by chemical oxidation of graphite oxide with a high number of acidic oxygen-bearing functional groups such as –COOH and –OH. Besides, the neutralized GQDs were also evaluated as an electrolyte in a solid supercapacitor. Neutralization of their acidic functional groups using KOH could improve the ionic conductivity and ion-donating ability of GQDs. As a result, the solid-state supercapacitor using GQDs as its electrolyte performed a specific capacitance of 42 F g^−1^ at a current density value of 0.1 A g^−1^ with excellent capacitance retention after 1000 cycles [120]. GQDs without any additional materials potentially improved SC performance by playing the role of an electrode in a micro-supercapacitor or double-layer supercapacitor and of an electrolyte in solid-state supercapacitor. Even though their performance indicators are still lower than those of composite structures, the advancements were expected to be notable.

## 5. Recent Progress of Carbon-Based QD Composites

In this part, we discuss the combination of C-QDs with other conventional SC materials to form composites. Most reports on C-QD-based SCs are composites of GQDs with conventional electrode materials, such as metal oxides, activated carbon, metal-organic framework (MOF), or polymer semiconductor materials, in order to enhance device performances. Recently, transition metals emerged as electrode materials for pseudocapacitance supercapacitors. They were selected because of their high theoretical capacity, vast properties variety related to toxicity, high conductivity, and broad voltage scan range [131].

### 5.1. Carbon Dot Composites

**Figure 7:**
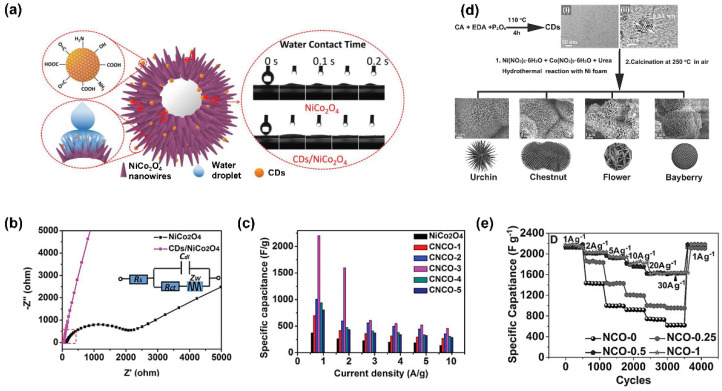
(**a**) Schematic illustration of the CD effect on NiCo_2_O_4_ hydrophilicity properties. (**b**) Nyquist plots of the NiCo_2_O_4_ and CDs/NiCo_2_O_4_ electrodes. (**c**). The specific capacitance of NiCo_2_O_4_ and CDs/NiCo_2_O_4_ in varying current densities: reproduced from Reference [132]. Copyright John Wiley and Sons, 2019. (**d**) The CDs/NiCo_2_O_4_ composites with different morphologies prepared using a uniform size of CDs. (i) The corresponding TEM image of the as-synthesized CDs and (ii) High-Resolution TEM image of the as-synthesized CDs. (**e**) Cycling stability and rate performance of CDs/NiCo_2_O_4_ composites at various current densities: reproduced from Reference [133]. Copyright John Wiley and Sons, 2016.

Carbon dots (CDs), as one class of C-QDs, are distinctive among other C-QDs. They have a lower degree of crystallinity. However, CDs have various emerging properties when combined with the other materials for SC applications. Initially, the use of carbon dots as tandem materials in SC aimed to exploit their surface functionalization to increase SC performances. As they have a lot of hydrophilic functional groups on the surface, CDs can increase the electrodes’ wettability as reported in their utilization together with NiCo_2_O_4_ nanowires, as shown in Figure 7a [132]. Furthermore, CDs also may increase the active surface area, provide unique morphology, and modify the conductivity. An equivalent circuit, as shown in Figure 7b, was selected to be fitted to the impedance data. A more vertical line at the low-frequency region was shown in a Nyquist plot of CDs/NiCo_2_O_4_, indicating easier diffusion of electrolyte ions to the CDs/NiCo_2_O_4_ and better electrochemical performance than a bare NiCo_2_O_4_ electrode. A remarkably high enhancement of the specific capacitance of CDs/NiCo_2_O_4-_ compared to NiCo_2_O_4_ was seen from 699 F g^−1^ to 2202 F g^−1^, as shown in Figure 7c.

Transition metal sulfides are among the common materials combined with CDs to co-enhance their properties. Combining nickel sulfide with CDs can be done by co-synthesizing them using the hydrothermal method [67]. The composite exhibited a supercapacitor with a specific capacity as high as 880 F g^−1^ and stable for 2000 cycles of the charge–discharge process. The incorporation of the CD in this NiS matrix improved the charge transfer process. CDs are also known to have hydroxyl and carboxyl groups on their surface. These functional groups create a negative charge on the surface [134].

The existence of CD in metal oxide and metal chalcogenides might significantly alter the structures. Ji et al. reported a flower structure of NiO/CDs after combining NiO with CDs. The porous structure is also formed from the synthesis process, which results in better penetration of the electrolyte into the electrode [135]. Others also reported that the presence of carbon inside the synthesis process when creating CD-containing composites also changes the structure of the materials [136,137].

CDs are reported not only to enhance the supercapacitor performances of metal oxide but also to play a role as a structural directing agent of NiCo_2_O_4_ composites [133]. By varying the concentration of CDs, the nanostructure morphology of NiCo_2_O_4_ changes significantly, as illustrated in Figure 7d. These distinguished morphologies provide the divergent type of specific surface area that results in distinct specific capacitance values of each composites as well as cycling durability, as shown in Figure 7e. The differences in morphological structures also might become the distinguished ion diffusion mechanism on the composite materials.

In general, adding carbon-derived materials in excess provides carbon matrices for the transition metal species. The CDs that act as matrices provide significant amount of positive charges that leads to higher capacitance in the composites [138]. Besides, In NiS/C-QD materials, the resistance of the material decreases from 150 Ω to only 75 Ω after the addition of CDs. Zhenyuan et al. reported another example of compositing with another transition metal sulfide, CoS. Compared to the bare CoS sample, CoS/N-doped CDs have a higher specific area. However, they found that excessive nitrogen doping into CDs can hinder contact between the electrolyte and electrode, creating another problem to be overcome, which is the decrease of specific capacitance [139].

On the other hand, Xiang et al. reported that compositing metal chalcogenides with CDs might increase the dielectric coefficient of the composites due to the formation of sandwich arrangements on CDs. The improvement in the dielectric coefficient can give us a benefit in increasing the EDLC performance. As mentioned in previous discussions, the capacitance of EDLC can be affected by the dielectric coefficient of the electrode materials. Observations of EDLC capacitance enhancement in GO/CDs/PPy can further investigate hybrid SCs, which combines EDLC and pseudocapacitive properties in a single material system [140].

Recently, Ravi et al. also reported a combination of graphene nanoplatelets, PPy, and carbon dots, resulting in a combination of EDLC and pseudocapacitance. The solid-state SCs utilized polyvinyl alcohol (PVA) as the electrolyte and GNP-CD-PPy as the electrode. The introduction of such a combination of CD and GNP on PPy to modify the surface of the eggshell membranes in this device brought a higher surface area. In this GNP-CD-PPy, it was concluded that the CDs contributed to elevating the capacitance to values above 80% of the original performance parameters [140,141].

The compositing CDs with a conductive polymer have the unique capability of creating flexible SCs, which is attractive for further development. The enhanced supercapacitive properties may originate from the addition of CDs to both the conductive polymer and the transition metal oxides/chalcogenides. We summarize the state-of-the-art of the reported C-QDs composite electrode materials in Table 2.

### 5.2. Carbon Quantum Dot Composites

C-QDs have been combined with many supercapacitor electrode materials to enhance their performances by modifying either their electrochemical properties or their surface properties. C-QDs have been reported to provide carbon matrices to improve their electrical conductivity and solid electrolyte interface stability [145]. Wei et al. reported that a combination of NaOH treatment and the addition of C-QDs into carbon matrices would give a large hierarchical structure which provides ion-buffering reservoirs resulting in better electronic properties.

**Figure 8:**
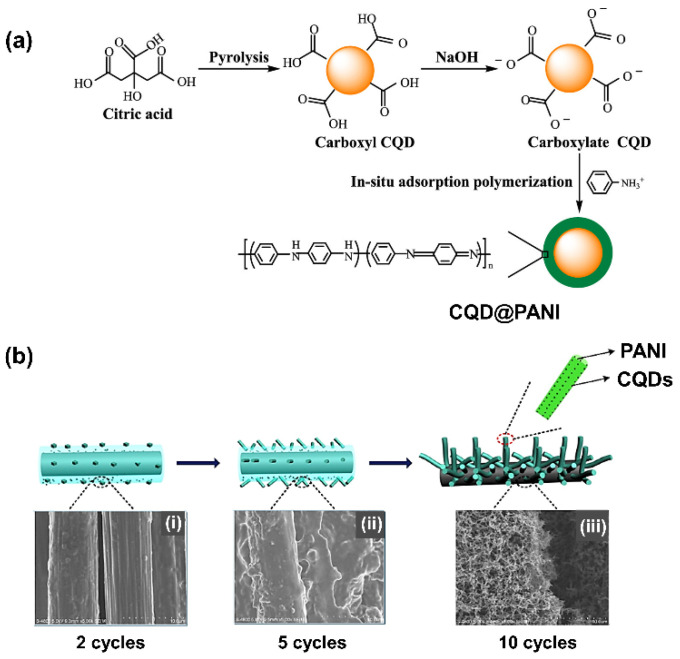
(**a**) Schematic illustration of the synthesis of core shells C-QDs@PANI by pyrolysis: reproduced with permission from Reference [149]. Copyright Elsevier, 2019. (**b**) Schematic illustrations and corresponding SEM images of the C-QDs-PANI/CFs(LI) prepared by cyclic voltammetry (CV) electrodeposition in varying cycles of 2, 5, and 10: (i) 2 cycles; (ii) 5 cycles; (iii) 10 cycles. Reproduced from Reference [162]. Copyright Elsevier, 2017.

In C-QDs/metal oxide, the C-QDs were utilized as bridges to connect oxide material domains. In MnO_2_ [148] matrices, the C-QDs not only improved the specific capacitance but also broadened the potential window of the composite for SC operation [92]. The other suitable electrode materials than can be combined with C-QDs are polymer semiconductors. It was proposed that the enhanced SC performances of C-QDs/conducting polymer electrodes correspond to the noncovalent approach between C-QDs and a conductive polymer [149]. There are three possible noncovalent approaches between the C-QDs and the polymer semiconductor: *π*−*π* stacking interaction, van der Waals interaction, or electrostatic interaction [162]. The formation of *π*−*π* conjugation between the polymer and C-QD surface provides significant conductive electron transfer due to the lower energy interfaces. Recently, the core-shell structure of C-QDs coated by the Polyaniline (PANI) polymer has been synthesized using the pyrolysis method (Figure 8a). The ratio between aniline and C-QDs was found to be one of the crucial parameters to demonstrate the highest specific capacitance using this composite. After 500 cycles, the C-QDs@PANI electrode maintained 87.7% of its initial capacity, implying better capacity retention than bare C-QDs or bare PANI electrodes [149]. Besides, fabrication of this C-QD composite using photo-assisted cyclic voltammetry (Figure 8b) also increased the contact conformation between the C-QD–PANI composite and the utilized electrolyte [162].

After compositing C-QDs with transition metal materials, the functionalization of C-QDs also lead to a better wettability of the electrode and superior electroactive surface and improved performance due to better surface properties [92,147]. The formation of rough or porous structures in the conductive polymer after adding C-QDs increased the surface-to-volume ratio of the materials. The wider surface area can provide extra ion storage, resulting in high capacitive performance. From structural perspectives, the structural enhancement in transition metal oxides by the introduction of C-QDs also facilitates higher electrical and modification of ionic movement for the composite [143,144].

Furthermore, in the vicinity of the interface between C-QDs/metal oxides in the composite, the valence and the conduction bands of both C-QDs and metal oxide bands generate an electrical field that can act as a potential barrier for electron transmission. This barrier can carry out partial potential and can impede the external electric field to broaden the potential window for SCs. Recently, Lv et al. reported the use of C-QDs as a composite material filler in MnO_2_/graphene-aerogel. The insertion of C-QDs in the graphene aerogel layer increased the specific surface area of the materials, producing a more accessible layer and preventing any agglomeration that would appear after charging. Also, the conductivity of this material was increased after the addition of C-QDs [148]. The origin of this increase in conductivity still needs to be further clarified. Nevertheless, the crystalline structure of C-QDs might not play any crucial role.

In the synthesis of a conducting polymer, the addition of C-QDs can play a significant role in the electropolymerization process and in forming a porous structure in C-QDs/PPy composites. With an increase in electrodeposition time, the “nano-island” of C-QDs/PPy gradually grows [150]. The hydroxyl groups on C-QD surfaces also have a role as electron donors and strong reductants [73,163]. Nevertheless, PPy as a conductive polymer still faces poor cyclability even after compositing with C-QDs. Therefore, further advancements are needed, such as through the addition of doping using other materials or modification of the C-QD composite using GQDs that provide better structural stability in PPy composites [159,164].

### 5.3. Graphene Quantum Dot Composites

Combining transition metals with sulfide is known to provide higher pseudocapacitive performance in SC applications compared to transition metal oxide. Nevertheless, they also suffer from the same problems that arise in transition metal oxides, such as low electron transportability and quick capacity decay at high charging–discharging rates. A combination of transition metal sulfide, such as MoS_2_, with GQD can lower resistance at the electrode and electrolyte interface, as reported by Monghimian et al. [82]. Moreover, the hollow structure that arises from proper synthesis of the GQDs, which is favorable for relaxing the volume expansion, can alleviate the potential structural damage during the cycling process [158].

**Figure 9:**
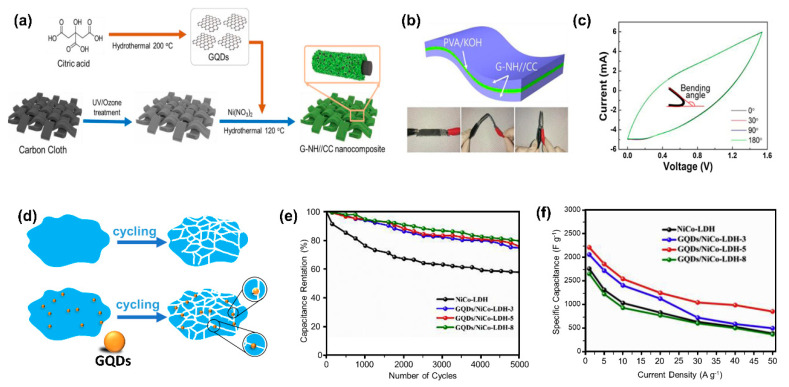
(**a**) Schematic illustration of the synthesis of GQDs/Ni(OH)_2_ nanocomposites on a carbon cloth by the hydrothermal method. (**b**) A symmetric supercapacitor of GQDs/Ni(OH)_2_ on a carbon cloth at different degrees of bending states. (**c**) The CV results of GQDs/Ni(OH)_2_ at different degrees of bending states: reproduced from Reference [153]. Copyright Elsevier, 2002. (**d**) the Role of GQDs as toughening materials in pseudocapacitors: GQDs also act as bridge of electron (**e**) capacity retention of various amounts of GQDs in nickel−cobalt Layered double hydroxide (LDH) over 5000 cycles at 20 A g^−1^. (**f**) Specific capacitance of all variations of GQD amounts in Ni−Co LDH: reproduced from Reference [100]. Copyright Elsevier, 2019.

It has been reported that the addition of GQDs on transition metal oxide (TMO) or transition metal sulfide (TMS) materials is mainly to increase the composite conductivity [152]. Figure 9a shows the addition of GQDs into Ni(OH)_2_/carbon cloth. This addition enhanced the conductivity performance of the SC electrodes and made it capable of operating for up to 2000 cycles. The enhanced electrical conductivity was thought to cause the formation of ohmic contact by Ni(OH)_2_ due to the addition of GQDs [153]. By the addition of GQDs, shown in Figure 9b, the electrodes are highly flexible. Further investigation of the GQDs/Ni(OH)_2_ are shown in Figure 9c, which tell us with the different bending angles that no significant changes on the CV was observed.

Wang et al. reported that coating Carbon Fiber Cloth (CFC)/PANI (polyaniline) alternatingly with GQDS/rGO, layer-by-layer, on a carbon cloth can demonstrate increased electrode performance [161]. In this case, the GQD’s role is to modify the hydrophobic nature of the carbon cloth, enhancing its interaction with the polymers. The PANI layer and GQD-rGO layer make strong electrostatic interactions between layers that cause the self-assembled 3D structures to have good structural stability. The capacitance value of (PANI/GQD-rGO)_20_/CFC remains at 89.7% with increasing current density, and it could be cycled at a higher rate compared to other variations that have similar initial capacitance. Nevertheless, as a pseudocapacitive material, PANI also has been known to have a problem in volumetric changes and internal stress during the charge–discharge process. It was the reason for a slight decrease in the capacitance of this type of material. Having the PANI embedded by GQD-rGO, internal stress during the charge–discharge cycle is effectively released. Consequently, together with high electrical conductivity and other improvements, PANI SCs show high specific capacitance (1036 F g^−1^) and superb cycling performance with a capacity retention of 97.7% after 10,000 cycles. This kind of enhancement by GQD composites is not only for pseudocapacitive materials but also for EDLC materials [161].

Exploitation of the GQD edge properties can be utilized to increase the SC properties of other materials. GQDs have abundant π-conjugation and edge sites, which make it possible to increase NiCO_2_O_4_ properties like conductivity, surface area, and wettability [157]. The unique homogenous edge states of GQDs can be the host sites for free electrons, for example, from transition metals in the corresponding composites. Free electrons accumulated at the edge can form an extra electrostatic attraction, which increase the specific capacitance of formed EDLs on the surface of the composite [156]. In conductive polymer materials, negatively charged GQDs/polymers could provide positive ions on the surface, giving a possibility to increase the specific capacitance from the EDLC phenomenon [160]. Li et al. reported that adding GQDs into a Metal-Organic Framework (MOF) enhances the wettability of MOF and increases its specific surface area. Consequently, its specific capacitance increases. N-GQDs/cMOF in an asymmetric supercapacitor can exhibit a high specific capacitance of 294.1 F g^−1^ at 0.5 A g^−1^. This high specific capacitance originated from the high electrochemical performance via the pseudocapacitive behavior of N-GQDs [121]. PEDOT:PVA is another example of a high conductive polymer, which was also combined with GQDs and demonstrated high SC performances. A core-shell structure that wraps GQDs using a PEDOT:PVA nanofiber was prepared using the electrospinning method. Using this method, the GQD particle size can be made smaller than the typical pristine size, thus being beneficial for supercapacitors [160].

The morphology of electrodes is among the crucial parameters for SC device performances. Adding GQDs into composite can induce morphological changes during the synthesis process. Huang et al. demonstrated it when adding GQDs into CuCo_2_S_4_. The addition of GQDs improved the morphology of CuCo_2_S_4_ to become more suitable for a supercapacitor device, which has a rougher surface. This morphological change enhanced the electrochemical capacitance by almost 89% from 908.9 F g^−1^ to 1725 F g^−1^, after adding GQD into CuCo_2_S_4_ [154]. The charge and discharge cycling performance of GQDs/CuCo_2_S_4_ show 90% capacity retention after 10,000 cycles. Furthermore, adding GQDs also improves CuCo_2_S_4_ conductivity. The fast electron-transfer rate caused significant enhancement in the electrochemical performance as measured by electrochemical impedance spectroscopy (EIS).

Adding GQDs during the synthesis process of the transition metal sulfide and polymer composite gives another beneficial effect to the synthesized materials. The GQDs can restrain the anisotropic growth of the transition metal sulfide and modify the polarity of the polymer, thus leading to nanosized clustering of the materials [160]. In line with the influence of C-QDs, the impact of GQD addition in the synthesis process also leads to the formation of a rough surface that results in better electrochemical performance. This effect is in addition to the role of C-QDs in providing better solubility. In the case of metal hydroxide, the addition of GQDs leads to its directional growth in the specific direction, thus producing morphologies desirable for better supercapacitors [155].

Another benefit of GQD usage in a composite is the formation of the M-O-C (M = metal) covalent bond at the interface of the GQD species and the metal oxide hosts [148,156,165]. MnO_2_/GQDs synthesized using the metallorganic chemical vapor deposition (MOCVD) method were reported to show M-O-C bonds, which became the origin of the increased capacity retention in the corresponding SC devices. Another report also revealed that the strong bond of M-O-C could act as new active sites for a redox reaction [165]. This kind of heterostructure possessing M-O-C bonds between a metal oxide and the C-QDs can be inherently beneficial for many aspects of supercapacitor electrodes [156]. Nevertheless, further study in varying the metal oxide species is still critical. In particular, some reports (e.g., Huang et al.) showed that adding GQDs did not change the morphology of NiMoO_4_ in the composite [154]_._ Therefore, we still need to establish some rules, since we need to consider that not all known supercapacitor electrodes are suitable for composition using C-QDs.

C-QDs can also be applied as toughening components in the SC electrode [100]. Figure 9d shows that GQDs were added to patch crack propagation paths, thus preventing failure in NiCo-layered double hydroxides (NiCo-LDH). It is proven that, after 5000 cycles, GQDs/NiCo-LDH still have 75.3% of its initial capacity, which is much higher than NiCo-LDH without inserted GQDs (remaining capacity at <60%) (Figure 9e). Besides, the addition of GQDs with the specified amount can increase the specific capacitance in NiCo-LDH to 25% compared to the bare one (Figure 9f). Additionally, GQDs can suppress dissolution and agglomeration of transition metal compounds and polymers, enhancing the electrode stability [158,159]. We summarized the performance comparisons of electrodes that combine transition metals or polymers with GQDs in Table 2.

## 6. Challenges and Future Perspectives

As reviewed above, C-QDs are promising candidates for the next generation of supercapacitors. Owing to its simple synthesis, high conductivity, and charge donor ability, some C-QDs have been successfully reported to be used as electrodes and electrolytes in SC devices. However, many substantial challenges still need to be considered and clarified in the near future to advance the research on C-QD-based supercapacitors. Among the identified challenges are the following:

-*Requirement for deep understanding of the charge storage mechanism in C-QD-based electrodes*. So far, the excellent SC performance by incorporating C-QDs that has been reported only focused on the use of C-QDs as the electron backbone for charge transport in the electrode. However, both systematic experimental and theoretical approaches should be conducted to elucidate the characteristics of the C-QD-based electrode/electrolyte interface, which are thought to be also crucial keys in controlling the charge storage and the delivery of charge. Understanding interfacial resistance between the C-QDs and the corresponding current collector is also an essential parameter that should be investigated.

-*Diversification of synthesis, purification, and functionalization routes of C-QDs*. Since C-QDs serve as an electric conductivity booster of many other electrode materials, the structure and morphology of C-QDs can strongly determine the architecture of the C-QD composite electrode. For example, a small dimension of the C-QDs can easily shorten the distance traveled by the ions that diffuse into the electrode from the surface. A well-monodispersed quantum dot with a high degree of crystallinity is preferable for the desired isotropic electrical conductivity characteristic. Therefore, effective synthesis routes and smart purification procedures to obtain these desired C-QDs are highly required. Moreover, surface functionalization of C-QDs also play crucial roles in designing the architecture of the composites, which warrant further optimizations.

*-Enhancement of the performance-to-fabrication cost ratio.* The global supercapacitor market was valued at $3.27 billion in 2019, and it is expected to grow annually by 23.3% and to reach $16.95 billion by 2027 [166,167]. Therefore, it is essential to further enhance SC performances based on all aspects, including specific capacitance, power density, energy density, and long-term cycling durability. Optimization of these performance parameters against the related fabrication cost should meet the commercial requirement of SC applications. Moreover, C-QD electrochemical properties are attractive for many applications beyond simple SCs, including some optoelectronic devices and the other SC-derived devices.

### 6.1. Wearable Supercapacitors

Now and in the near future, energy storage devices should fit and conform to different structures and shapes (e.g., human/animal bodies, plants, and soft robots) since applications will not only be limited to a planar place. Flexible and conformal supercapacitors are among the most demanded applications. With this ability, fabrics that can store energy can be developed. On the other hand, bio-interfaced IoT sensors can also benefit from the development of these flexible and conformal supercapacitor devices. Material-wise for biocompatibility, carbon-based supercapacitors are the leading candidate for such applications.

There are various properties of C-QDs that made them remarkably attractive for applications. First, the C-QDs structure allows us to bend our supercapacitor while maintaining the electrochemical properties. As shown in Figure 9b,c, which was the work of Hong showing GQDs/Ni(OH)_2_ on carbon cloths, a composite for a flexible supercapacitor electrode [126,153]. This composite structure allows the supercapacitor to bend up to 180° with almost no significant change on the CV curve. However, to prove that supercapacitors are flexible, CV measurement in different bending states are crucially needed in this field. The C-QD matrix in composites allows some other material combination also to have good flexibility. Recently, several composites of C-QD materials have demonstrated outstanding flexibility with excellent bending capabilities of more than 90° but without changing the electrochemical performance after bending [151,153]. Testing the durability of the electrochemical properties of SCs while changing the bending angle is a crucial test to prove the wearability of C-QD SCs.

**Figure 10:**
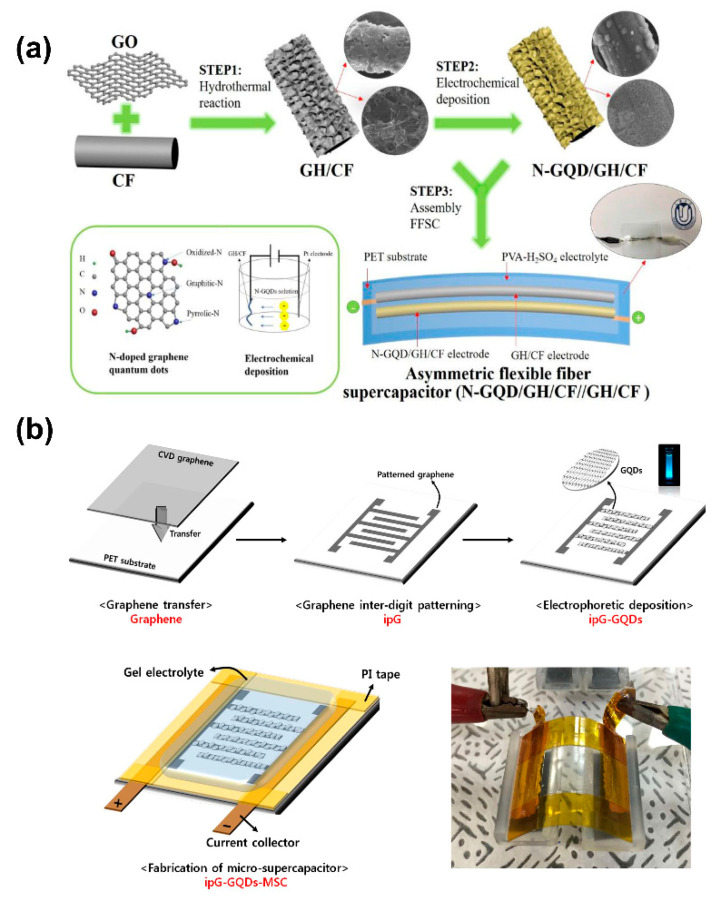
(**a**) The synthesis process of N-GQDs on carbon fiber/graphene hydrogel with a hydrothermal reaction followed by electrodeposition: the symmetric supercapacitor was fabricated on a flexible substrate and PVA/H_2_SO_4_ to ensure the flexibility, portability, and stability. Reproduced from Reference [122]. Copyright Elsevier, 2019. (**b**). Schematic illustration of ipG-GQDs-MSC fabrication starting from chemical vapor deposition (CVD) onto a polyethylene terephthalate (PET) surface, patterning graphene to create interdigitated electrodes: the GQDs are deposited by using electrophoretic. Reproduced from Reference [170]. Copyright Elsevier, 2016.

In this kind of flexible supercapacitor, a gel electrolyte is commonly chosen to prevent leakage during the bending test. Among the examples, Zhen et al. used PVA-H_2_SO_4_ as the electrolyte in an N-GQD/GH/CF as an asymmetric flexible fiber supercapacitor, which was synthesized using the hydrothermal method followed by electrochemical deposition (Figure 10a). The flexible supercapacitor device assembly was built on polyethylene terephthalate (PET) as the substrate [122].

Carbon cloth [153], conductive polymers [168], and nickel foam [169] are commonly selected flexible electrodes for C-QD SC devices to satisfy the bending capability. Keunsik et al. proposed another alternative by fabricating micro-supercapacitors based on interdigitated graphene for micro-supercapacitors (ipG-GQDs-MSC) (Figure 10b). The micro-supercapacitor showed no significant changes in electrochemical performance. However, it still suffered a capacity decrease after charge–discharge cycling while binding [170]. Furthermore, it is in the interest of applications to have SCs with a transparent electrode to provide additional value to wearable devices. C-QDs are highly transparent materials; thus, when implementing such a possibility, a highly transparent SC is foreseeable.

There are several routes to fabricate flexible and wearable supercapacitor devices. Singh et al. reported the use of electrospinning to create flexible Cu@Ni@NiCoS nanofiber SCs [171]. As seen in Figure 10a, by inserting a carbon cloth as the electrode, GQDs/Ni(OH)_2_ nanocomposite can be deposited on the carbon cloth, creating a flexible electrode [153]. This C-QD can be synthesized using the hydrothermal method because of its simple mechanism that allowed a one-step synthesis route to produce a robust SC electrode.

### 6.2. Quantum Capacitance Supercapacitor

Another emerging outlook on how to significantly enhance the supercapacitor capacity in nanostructured materials, including C-QD, is by looking into possibilities to exploit the quantum capacitance effect. Quantum mechanics governs physical phenomena in nanoscale. Thus, complementing our understanding of the working mechanism with quantum mechanics insight would give us a whole new perspective in designing materials for supercapacitors, particularly those involving nanomaterials. The material’s capacitance microscopically and strongly depends on the so-called electronic density-of-states (DOS) of the materials. In classical 3-dimensional bulk materials, the DOS for each electronic state is continuous. In this case, the geometric capacitance is much more dominant in limiting the capacitance value rather than filling these continuous energy levels.

Electronic quantum confinement in 0D, 1D, and 2D materials significantly changes the corresponding electronic DOS. In 2D materials, there are energy states where the DOS abruptly changes. On the other hand, 1D and 0D materials in some energy values can have distinguished high DOS values in the form of van Hove singularity or the formation of discrete energy levels, respectively. The filling of these DOS features by having the Fermi level shift in the capacitor operation to reach those electronic energy levels can significantly enhance the capacitance. In some regimes, this so-called quantum capacitance may overarch the geometrical capacitance [172]. Mathematically, in a system where quantum capacitance might need to be taken account, the contribution of quantum capacitance to the total capacitance of the supercapacitor can be rewritten as follows [173]:

(3) $$\frac{1}{C_{tot}}=\frac{1}{C_{G}}+\frac{1}{C_{Q}}$$

The additional terms $C_{Q}$ strongly depends on the configuration of the Density of States (DOS) of the materials. The theoretical formalism in approximating the quantum capacitance in 1D or 2D nanomaterials can be seen in the following equation.

(4) $$C_{Q}=\frac{q^{2}}{2\mathrm{kTh}}\sqrt{\frac{m}{2}}\int_{0}^{\infty }\frac{v\left(E\right)}{\sqrt{E}}\left[\begin{array}{c} {\sec h}^{2}\left(\frac{E+\frac{E_{G}}{2}-qV_{a}}{2kT}\right)\\ +\;{\sec h}^{2}\left(\frac{E+\frac{E_{G}}{2}+qV_{a}}{2kT}\right) \end{array}\right]$$

with v(E) as the number of contributing bands at a given energy and $V_{a}$ as the local electrostatic potential [174]. Experimentally, a strong influence of quantum capacitance of 1D materials and 2D materials have been reported in both carbon nanotubes and graphene, respectively [69,175].

**Figure 11:**
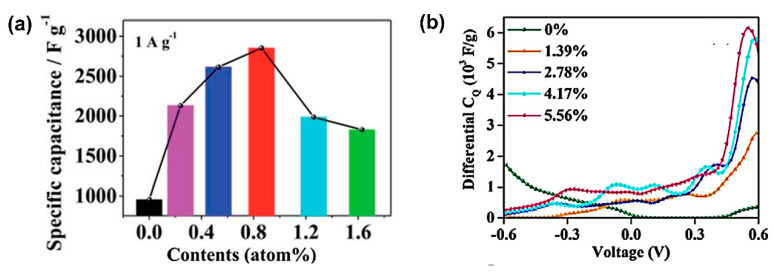
(**a**) The specific capacitances for CuS:Ce^3+^ with different Ce doping concentrations at a current density of 1 A g^−1^: reproduced from Reference [180]. Copyright The Royal Society of Chemistry, 2019 (**b**) The calculated C_Q_^dif^ of pristine and N−doped graphidyne at site II under different doping concentrations as a function of local electrode potential: reproduced from Reference [181]. Copyright Elsevier, 2020.

Besides having the quantum capacitance effect arising from electronic confinement due to low dimensionality, some researchers approached the possibility by engineering the band structure of materials, such as introducing dopants, functional groups, or defects, to create concentrated electronic density of states in one particular electronic state. These attempts can be seen in graphene [176,177]. Furthermore, this kind of dopant effect also recently has been studied for carbon-based material, resulting in an almost similar quantum capacitance phenomenon on the materials [178,179]. Chuai et al. reported that, by doping rare earth elements into CuS nanocrystals, the supercapacitor performance was remarkably enhanced. The supercapacitor specific capacitance increased by three times to 2853 F/g by adding 0.8wt% cerium (Figure 11a) [180]. This trend implies that, in the future, C-QDs can also be exploited in such a way.

Currently, theoretical calculations predicting the quantum capacitance capability of materials are actively being explored using DFT studies. From an investigation of the DOS of the materials and the influence of the dopants, the potential quantum capacitance can be predicted. In graphidyne (Figure 11b), it was predicted that doping various amounts of nitrogen and boron dopants into different sites can tune the quantum capacitance of materials [181]. This result is in line with other predicted increases of quantum capacitance in MoS_2_ after introducing dopant in the thin film [182]. So far, to the best of our knowledge, there is still no theoretical calculations for C-QD supercapacitors with enhancements that facilitate quantum capacitance. Nevertheless, the influence of quantum capacitance existence on graphene-based supercapacitors has been reported [183,184]. Therefore, an evaluation of the quantum capacitance effect in C-QD-based supercapacitors is foreseeable as a new way to realize high-performance supercapacitors.

### 6.3. Self-Charging Supercapacitor

The charging mechanism of a supercapacitor allows us to charge it with abundant energy sources available in nature, like light, thermal, vibration, or even gravitational energy [185,186,187,188]. In the thermal harvesting device family, the potential to have a Soret effect occurring in the electrolyte of the supercapacitor is attractive since it can be compared with having a high Seebeck coefficient in a thermoelectric device. The Soret effect dictates that temperature differences generate an ion concentration gradient due to thermo-diffusion on ions in the electrolyte [189,190]. Consequently, these ions can accumulate at the interface of the electrolyte/electrode as in a supercapacitor. In the Soret effect, connecting to an external load is not as easy as in traditional thermoelectricity due to the blocked thermo-diffusion ion on the metal surface. Recently, Zhao et al. proposed using a CNT on the electrode material to harvest the Soret effect by utilizing the EDLC charging principle of the CNT [191]. The concept of optimizing charging of the CNT EDLC by the Soret effect can be divided into two parts: selection of the ionic electrolyte to harvest the thermal energy and design of the supercapacitor electrodes. CNTs were used for their suitable properties as a supercapacitor electrode. They are also considerably electrochemically stable over a large potential window created by the Soret effect [191,192].

**Figure 12:**
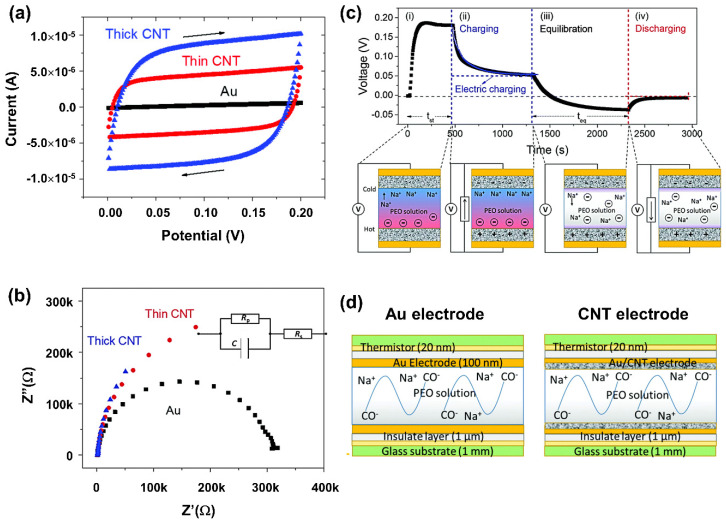
An SC’s self−charging demonstration utilized by the Soret effect: (**a**) CV curves from different types of electrodes in a square shape indicating the purely capacitive behavior of an electrode, with thick CNT results showing higher capacitance; (**b**) a Nyquist plot from impedance spectroscopy measurement of Au, thin CNT, and thick CNT electrodes, with equivalent circuit parameters the capacitance of thick CNT being higher; (**c**) a charge−discharge cycle of an Ionic Thermoelectric Supercapacitor (ITESC) establishing a temperature gradient of 16 K and discharging after the equilibration phase; and (**d**) a schematic illustration of the ionic thermoelectric supercapacitor device configuration with two different electrodes (right: Au and left: CNT) and with the reaction that takes place in the solution: reprinted from Reference [191]. Copyright. The Royal Society of Chemistry, 2016.

So far, as measured by EIS and CV (Figure 12), the performance of the CNT supercapacitor in the abovementioned integrated device is still lower than the state-of-the-art of C-QD electrochemical supercapacitors. Therefore, there is a high chance that one can significantly improve the Ionic Thermoelectric Supercapacitor (ITESC) performance in the near future by changing the CNT to C-QDs for the SC electrodes, besides performing some other investigation related to electrolyte use. Some other researchers have also reported the use of CNT in combination with PANI for a thermally rechargeable solid-state supercapacitor. With the use of different electrolytes, the charging of CNT/PANI electrodes can also be tuned, showing the possibility to use many other types of materials for the ionic thermoelectric part of SC electrode materials. A small temperature gradient of 5K can produce up to 38 mV open-circuit voltage with areal capacitance about 1200 F cm^−2^ in CNT/PANI ITESC after thermal charging is completed [193].

Another unexpected energy source that may self-charge the supercapacitor is gravitational energy. Dan et al. recently reported the gravity-induced self-charging of a CNT/PANI supercapacitor [186]. The mechanism of the potential buildup is due to the directional ionic movement by gravity or any other exerted mechanical forces. After continuously rotating the device in 1000 rpm for 420 s to create a centrifugal force, the potential difference in buildup reached over 60 mV. Some other example in which potential voltage built up due to ion movements by gravity/forces that can be used to charge supercapacitors directly was also recently reported on 2D materials, such as graphene or MoS_2_ surfaces [194]. These phenomena open up ideas on how to further utilize supercapacitors and to develop many other applications surrounding this device.

## 7. Conclusions

In this review, we summarized that enhanced SC performances could be achieved by introducing C-QDs (in form of CDs, C-QDs, or GQDs) as electrode or composite for SCs. So far, the focus of the C-QDs role in SC is mainly in the electrode of double-layer SCs and electrodes in pseudo-supercapacitors. Nevertheless, recently, some reports also started demonstrating the incorporation of C-QDs in the electrolyte of solid-state SCs. Elaboration of the designs and structures of the incorporated C-QDs with other electrode materials has provided promising strategies for practical SC application and future research. Although the research on C-QDs for SC application is still at its early stage, it implies that many avenues have not been explored yet. The chances to use C-QDs, either in their bare material forms or in composite forms (e.g., together with TMO or TMS), are still plentiful.

Furthermore, optimizing the combination of relevant EDLCs and pseudocapacitance to obtain greater specific capacitance by these materials will be one of the possible endeavors. In particular, C-QDs can also act as quantum confined materials, in which energy bands with a large density of states can become accessible, thus opening possibilities to store more charges due to the quantum capacitance effect. Complete understanding of the relationship between material design and the formed quantum capacitance would be vital for developing high-performance supercapacitors based on nanomaterials, including C-QDs. Soon, ubiquitous flexible and conformal supercapacitors based on C-QDs might become a viable energy storage device for many applications. Nevertheless, there are still many issues to be resolved for their further development related to the ultimate performance to be achieved, to sustainability of the fabrication process and utilizations, as well as to interfacing with other electronic devices. To resolve those issues, comprehensive evidence and convincing explanations on the electron transport and charging mechanism of C-QDs need to be clarified. Crystallinity, size, surface functionalization, as well as doping influence in various classes of C-QDs should be well understood so that one can control their electron transport. In other aspects, more standardized measurements to evaluate C-QD supercapacitor performance are crucially needed for reliable comparison.

## Figures and Tables

**Table 1:** Summary of various bare C-QD electrodes or combination with other carbon-based materials. NP = Not Published.

Material	Size (nm)	Initial Capacity	Current Density	Capacity Retention	Power Density	Energy Density	Ref
Bare GQDs	1–5	296 F/g	1 A/g	97.6% (5000×)	10,000 W/kg	22.2 Wh/kg	[119]
CDs/Graphene	2–5	91.9 F/g	0.1 A/cm^2^	96% (10,000×)	15 mw/cm^2^	46.67 μWh/cm^2^	[115]
ONCDs/Porous Hydrogels	3–10	483 F/g	1 A/g	100% (10,000×)	11,000 W/kg	13.5 Wh/kg	[124]
N-GQDs/Carbon Fibre/Graphene Hydrogel	1.5–7	93.7 F/cm^3^	20 mA/cm^3^	84.2% (10,000×)	200 W/kg	20.5 Wh/kg	[122]
GQDs/Carbon Cloth	10–20	77.5 mF/cm^2^	0.2 mA/cm^2^	NP	0.288 W/cm^2^	24,8 mWh/cm^2^	[125]
GQDs/Activated Carbon	2.9	388 F/g	1 A/g	100% (10,000×)	12.500 W/kg	7.99 Wh/kg	[123]
N,O-GQDs/CNT/Carbon Cloth	4–5	461 F/cm^3^	0.5 mA/cm^2^	87.5% (2000×)	0.1 mW/cm^2^	41.7 μWh/cm^2^	[126]
Amine-enriched GQDs	2.9	595 F/g	1 A/g	90% (10,000×)	250 W/kg	21.8 Wh/kg	[93]
S-GQDs	20	362 F/g	5 mV/s	NP	NP	NP	[47]
GQDs/Ultra microporous Carbon	2.96	270 F/g	1 A/g	100% (50,000×)	70 W/kg	9.38 Wh/kg	[127]
GQDs/3DG	NP	242 F/g	1.17 A/g	93% (10,000×)	NP	NP	[128]
GQDs/Conductive Porous Carbon	NP	315 F/g	1 A/g	100% (10,000×)	247.75 W/kg	9.21 Wh/kg	[129]
GH-GQD	8–10	451.7 F/g	0.5 A/g	89% (10,000×)	NP	NP	[130]

**Table 2:** Summary of various C-QD composites with their performance parameters for supercapacitor application (M = Transition Metal Composite, C = C-QDs, NP = Not Published).

Material	C-QDs Type	Size (nm)		Initial Capacity (F/g @ 1 A/g)		Capacity Retention		Power Density (W/kg)	Energy Density (Wh/kg)	Ref.
		M	C	without C-QDs	with C-QDs	without C-QDs	with C-QDs			
NiCo_2_O_4_ nanowire	CDs	100	2.56	699	2202	NP	95.3% (1100×)	499.8	73.5	[132]
Polypyrrole	CDs	NP	8.7	267	563	82.9% (1000×)	97% (1000×)	250.1	30.1	[140]
GNP-PPy	CDs	NP	2.9	NP	173 mF/cm^2^	NP	96% (2000×)	NP	NP	[141]
CoS/rGO	CDs	NP	NP	NP	697	NP	85.9% (10,000×)	16.000	36.6	[139]
NiCo_2_O_4_	CDs	20–2000	2–4	NP	2168	NP	99.96% (5000×)	216	62	[133]
V_2_O_5_	CDs	1000	200–220	60	270	67% (5000×)	87% (5000×)	4100	60	[137]
CuS	CDs	300	4	NP	736.1	NP	92% (5000×)	NP	NP	[142]
NiO/Co_3_O_4_	CDs	NP	NP	NP	1775	NP	95.7% (10,000)	16000	21.3	[135]
Bi_2_O_3_	CDs	NP	NP	849	1046	NP	85.3% (1500×)	770.9	79.9	[136]
NiCo_2_O_4_	C-QDs	250	3–6	NP	856	NP	98.7% (10,000×)	10240	27.8	[143]
RuO_2_	C-QDs	50–100	< 10	642	594	39.6% (5000×)	95.9 (5000×)	NP	NP	[144]
Bi_2_O_3_	C-QD	NP	NP	NP	125	NP	~97% (2500×)	8400	32	[145]
NiOH	C-QD	NP	NP	1414.3	2750 @20 A/g	26% (2000×)	64.1% (2000×)	683.7	57.4	[146]
MnO_2_ nanowire	C-QDs	1–4	NP	232	340	61.1% (10,000×)	76.4% (10,000×)	10000	12.5	[92]
Co_3_O_4_	C-QDs	9	3	1446	1603	96% (2000×)	97.6% (2000×)	810	78.4	[147]
MnO_2_	C-QDs	NP	3	527	721	70.3% (100,00×)	92% (10,000×)	1000	38.2	[148]
NiS	C-QDs	800	1–2	710	880	NP	NP	33000	30	[67]
PANI	C-QDs	4	10	182.5	222.7	~95% (500×)	~90% (500×)	NP	NP	[149]
Polypyrrole	C-QDs	NP	NP	215.6	424.6	NP	85.7% (2000×)	NC	NC	[150]
CuCo_2_O_4_	C-QDs	3	NP	464.4	779.8	NP	86.4% (5000×)	1203.7	39.5	[151]
Halloysite Nanotube	GQDs	NP	5–10	NP	338	NP	88% (5000×	230	50.03	[152]
Ni(OH)_2_	GQDs	NP	7	NP	1825	55.2% (8000×)	83.5% (8000×)	2021	80.8	[153]
CuCo_2_S_4_	GQDs	NP	NP	908	1725	NP	90% (10,000×)	NP	NP	[154]
MnCo_2_O_4.5_	GQDs	~100	NP	368	1625	NP	77% (5000×)	2800	24	[155]
MnO_2_	GQDs	10	1	400	1170	43.1% (10,000×)	92.7% (10,000×)	12351	118	[156]
MOF	GQDs	NP	3	188	780 @10 mV/s	NP	80.2% (10,000×)	5,000	6.81	[121]
MoS_2_	GQDs	NP	15	100	323.5	NP	92.3% (500×)	489.96	38.47	[82]
NiCo-LDH	GQDs	NP	2	1769	2220	55.7% (10,000×)	75.3% (10,000×)	8000	50.84	[100]
NiCo_2_O_4_	GQDs	800	2–4.5	1190	1382	99% (4000×)	99% (4000×)	800	38	[157]
NiCoS	GQDs	200	NP	562	678 @0.2 A/g	70% (5000×)	94% (10,000×)	NP	NP	[158]
Polypyrrole	GQDs	NP	5	437	560	~37% (1000×)	60.5% (1000×)	NP	NP	[159]
PVA-PEDOT	GQDs	NP	55.6	161.48	291.6	64% (1000×)	98% (1000×)	NP	16.95	[160]
PANI/rGO/CFC	GQDs	NP	NP	NP	NP	78% (10,000×)	97.7% (10,000×)	424	34.2	[161]

## Data Availability

Data sharing not applicable.
